# Economic policy and psychological violence: the hidden costs of Spain’s minimum wage reform

**DOI:** 10.1007/s13209-025-00324-x

**Published:** 2026-02-03

**Authors:** Yolanda F. Rebollo-Sanz, Núria Rodríguez-Planas

**Affiliations:** 1 Universidad Pablo Olavide, Seville, Spain; 2 Institut d’Economia de Barcelona (IEB), Universitat de Barcelona and City University of New York (CUNY), Queens College-CUNY, 300A Powdermaker Hall, 65-30 Kissena Blvd., Queens, NY 11367 USA

**Keywords:** Minimum wage increase, Doubly robust difference-in-differences, Intimate partner violence, Psychological violence, Household bargaining, J16, J12, J38, I8

## Abstract

This paper examines the impact of a 22% minimum wage increase in Spain on January 2019 on intimate partner violence using a doubly robust difference-in-differences strategy with inverse probability weighting and the nationally representative Spanish Survey of Violence Against Women. We find no effect of the reform on physical or sexual violence. However, treated women—those with a high predicted probability of working at minimum wage jobs—experienced a 42% increase in psychological violence. Labor market analysis of survey respondents reveals that the reform led to a substitution away from female employment toward her partner’s employment, reducing women’s bargaining power within the household. For women whose partner is five years older, the increase in violence is not accompanied with lower female labor market engagement, providing evidence of alternative mechanisms, such as disrupted gender roles, or instrumental violence. These findings highlight unintended consequences of wage policy and highlight the need for complementary policies and services addressing the dangers of gender-based and domestic violence.

## Introduction

Gender-based violence (GBV) remains a pervasive human rights crisis globally, as emphasized by United Nations Secretary-General António Guterres (2020). In the European Union (EU-27), recent data from the Fundamental Rights Agency (2024) show that 30.7% of women report having experienced physical or threats and/or sexual violence in their lifetime. Intimate partner violence (IPV), in particular, is alarmingly widespread: 17.7% of women have experienced physical or sexual violence from a partner, rising to 31.8% when psychological abuse is included, and 5.3% report recent victimization within the past year. Spain reflects these concerning trends, with national statistics reporting comparable prevalence rates of 14.4%, 28.6%, and 4.1%, respectively.

Beyond its devastating personal toll (World Health Organization 2013; Adams et al. 2013; Browne et al. 1999; Lloyd and Taluc 1999), IPV imposes significant economic and societal costs. The European Institute for Gender Equality (2021) estimates the annual cost of violence against women in the EU-27 at €290 billion, with IPV accounting for €152 billion. As a result, combating gender-based and domestic violence is a core priority of the European Commission’s Gender Equality Strategy 2020–2025.

This paper investigates whether a significant minimum wage increase can reduce the prevalence of IPV. We exploit a quasi-experimental labor market intervention: a 22% increase in Spain’s national minimum wage (from €736 to €900), implemented on January 1, 2019. Using nationally representative data from the Spanish Survey on Violence Against Women (SVAW), we assess the impact of this wage reform on women’s risk of experiencing IPV in the past 12 months.

Given the survey’s lack of information on earnings, we construct a predictive model for minimum wage employment based on socio-demographic characteristics using the *Muestra Continua de Vidas Laborales* (MCVL) that we apply to generate predicted probabilities of treatment for individuals in our SVAW survey. We then stratify women by their predicted probability of being affected by the reform and compare victimization trends before and after its implementation across high- and low-probability groups. We use doubly robust difference-in-differences (DiD) with inverse probability weighting (IPW) estimation (Sant’Anna and Zhao 2020) to ensure consistent estimation of the average treatment effect on the treated (ATT) under certain assumptions. This framework improves covariate balance and mitigates selection bias while accounting for unobserved time-invariant heterogeneity through the DiD structure. By comparing IPV victimization risks between these groups before and after the policy reform, we aim to better understand the potential mechanisms through which minimum wage increases among low-income earners might influence domestic violence dynamics in Spain. Using a minimum wage employment predictive model to proxy eligible workers reflects a substantial improvement over the standard practice in the literature, which often uses education level attainment (e.g., “no college degree”) as a proxy of eligible low-income workers.^1^

Theoretical frameworks—such as household bargaining, the backlash model, or the instrumental model—typically rely on changes in *relative* earnings between partners. While the minimum wage reform we study applied broadly to both men and women, recent evidence using administrative social security data reveals gender-differentiated effects stemming from the structure of the Spanish labor market as the reform disproportionately affected women: in 2018, 57.4% of minimum wage workers were female (Elias and Riudavets-Barcons, 2023). Similarly, Gorjón et al. (2024) find that the 2019 minimum wage increase disproportionately affected women in Spain.

According to the household bargaining model, a minimum wage increase would reduce IPV if women’s employment remains unaffected, as higher earnings would strengthen their bargaining power within the household given the gender-differentiated effects of the Spanish 1999 minimum wage increase. Conversely, if the reform displaces women from employment, the model would predict an increase in IPV due to a weakening of their bargaining position. A similar increase in IPV risk is predicted by models in which men use violence as a strategic tool to influence intra-household decision-making or to extract resources from their partners (Bloch and Rao, 2002; Bobonis et al. 2013; Eswaran and Malhotra 2011) as long as women’s employment is not negatively affected by the minimum wage reform and hence her relative earnings within the household increase after the reform. Similarly, the male backlash model would also anticipate an increase in IPV, as male partners may resort to violence to reassert dominance and restore traditional gender roles threatened by their partner’s improved economic position. In the case where the male partner was also a minimum wage earner, the reform would represent an absolute increase in household income with no relative change as long as both partners work the same number of hours, or a relative decline in women’s income within the household if she works less hours than him. Regardless, an absolute increase in household income would reduce financial stress, which is known to reduce the risk of violence.

Our empirical findings reveal that the Spanish 2019 minimum wage reform did not reduce partner violence. Furthermore, we find evidence that the minimum wage increase was associated with a 40% increase in reported partner violence, driven by psychological abuse. In addition, among SVAW respondents, the reform marginally reduced female employment while increasing male partners’ employment suggesting a substitution in employment among partners, away from the secondary earner. There is no evidence that the reform impacted marriage or couple formation or dissolution. These estimates point to a household bargaining model in which women’s lower economic bargaining power increases their risk of psychological victimization. In addition, there is evidence of alternative mechanisms, such as disrupted gender roles, or instrumental violence, for women whose partner is five years older as, in this case, the increased psychological victimization is not accompanied with lower female labor market engagement.

This paper is closely related to a nascent literature on the impact of earned income on IPV in advanced economies. Overall, this literature is supportive of the household bargaining model in which partners’ relative bargaining power allows them to negotiate outcomes that better align with their preferences. Hence, to the extent that the woman’s relative economic position within the household improves, she is more able to negotiate a lower level of violence by adopting economic or social sanctions against potentially abusive partners and/or threatening to terminate the relationship. In such type of economic model, also consistent with feminist theory, female economic empowerment—such as higher earnings and/or stronger labor market attachment—enhances woman’s outside options and reduces her risk of victimization by her partner. Exploiting county variation in the sex composition of the industrial structure in the state of California, Aizer (2010) estimates that the decline in the gender wage gap observed in California between 1990 and 2003 explains about 9% of the reduction in female hospitalizations for assault. Exploiting the expansion of the earned income tax credit after 1993, Cesur et al. (2025) find that the expansion of the EITC in the U.S. under OBRA-93 led to a 41% reduction in physical or sexual IPV among unmarried non-White women. In Europe, Anderberg et al*.* (2016) develop a model and find evidence from the British Crime Survey that higher male unemployment is associated with lower IPV, while the opposite is true for higher female unemployment.

Interestingly, empirical evidence from the Spanish context reveals a more complex relationship between women’s higher economic empowerment relative to their partners and their risk of victimization. Alonso-Borrego and Carrasco (2017, 2018, 2023) provide evidence supporting the household bargaining model in couples where both partners are employed or only the male partner works. Their research also identifies cases of instrumental violence by unemployed male partners consistent with theoretical frameworks such as backlash theory, and the extractive theory.

Tur-Prats (2021) further explores the relationship between partners’ unemployment and IPV in Spain, leveraging historical differences in gender norms across provinces. In line with the backlash theory, she finds that a decrease in female relative to male unemployment increases IPV, particularly in provinces with more traditional gender norms^2^—such as financial extraction or coercive control—as primary drivers of violence in these contexts. More recently, Arenas-Arroyo et al. (2021) also find evidence consistent with the backlash theory in Spain during the COVID-19 pandemic, showing that the risk of IPV increased in households where the male partner’s relative economic position had deteriorated.

By providing quasi-experimental evidence on the effects of a 22% increase in the Spanish minimum wage on IPV and identifying the mechanism driving this increase in violence, our study makes a significant contribution to the growing literature on the complex interplay between labor market interventions, household economic dynamics, and gender-based violence. Studying a large-scale wage reform, we offer novel insights into how policies aimed at improving economic outcomes can yield unintended social consequences. Our findings underscore the nuanced effects of wage policy on intimate partner relationships. These results highlight the importance of complementing economic reforms with targeted, gender-sensitive policies to mitigate adverse effects—particularly in vulnerable households where shifts in economic power may reduce the relative labor market involvement of the secondary worker and hence, her bargaining power, subsequently triggering conflict. In doing so, our work deepens the understanding of how structural economic changes reverberate through intimate and social domains.

Section 2 presents the minimum wage reform, followed by Sect. 3 with detailed information on the different datasets used for the analysis. Section 4 describe the methodological approach, Sect. 5 presents the main results, and Sect. 6 identifies the underlying mechanisms behind our results, prior to conclude in Sect. 7.

## Institutional context: minimum wage in Spain

In Spain, the Ministry of Employment and Social Security sets the minimum wage at daily, monthly, and annual levels. Adjustments to the minimum wage do not follow a specific formula, nor are they directly tied to changes in the consumer price index (CPI). However, these adjustments traditionally occur in January, apply nationally, and are influenced by various factors, including the CPI growth, national productivity, and employment levels.

**Fig. 1:**
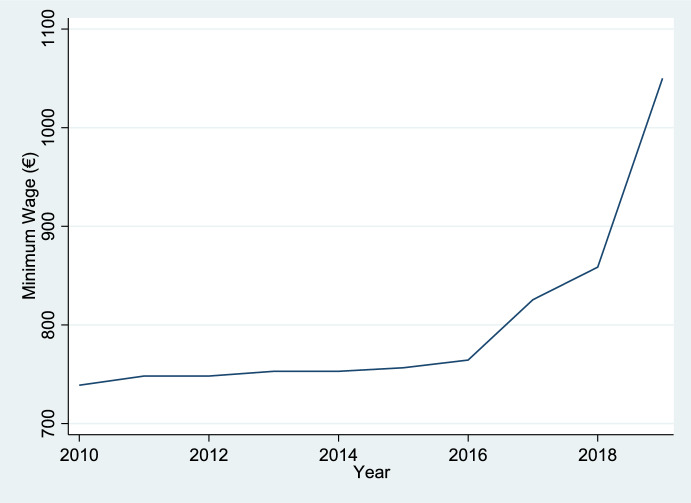
Minimum—Monthly Wage in Spain (2010–2019) *Note:* Monthly Nominal Minimum Wages (12 monthly payments). Spanish Official State Gazette (BOE). Minimum interprofessional wage. Royal Decrees establishing minimum wages for 2010–2019. Madrid: Official State Gazette

Prior to the 2019 minimum wage reform, the minimum wage remained relatively stable, hovering around the 600 euros per month (based on 14 annual payments^3^) for an extended period. It first reached the 600-euro threshold in 2008 and only rose modestly to 655,20 euros in 2016. In 2017 and 2018, the minimum wage increased by 8% and 4%, reaching 707,70 euros and 735,90 euros, respectively. However, it is not until 2019, that the monthly minimum wage experienced a significant increase to 900 euros. This 22% hike sharply contrasted with the minimal changes observed over the preceding decade, as illustrated in Fig. 1.

**Fig. 2:**
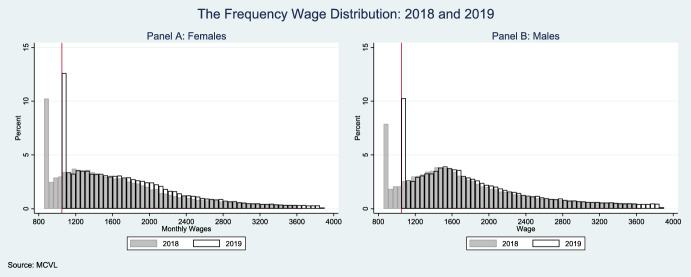
Impact of the Minimum Wage on the Working Population by Gender Note: Data from the Spanish Social Security panel records (CSWL). Figures show monthly wage distribution (in euros) for female workers (Panel A) and male workers (Panel B) in 2018 and 2019. The vertical red line indicates the new minimum wage threshold (900€) introduced in January 2019

Using data from the Spanish Social Security records (MCVL), we observe that the share of workers earning exactly the minimum wage increased from 12.2 to 15.5% after the reform. The impact was particularly pronounced when analyzed by gender, with female workers being more affected than their male counterparts. As illustrated in Fig. 2, which shows the monthly wage distribution^4^ by gender before and after the reform, the density of workers at the new minimum wage threshold (900€) is notably higher for women, with 18% of female workers being affected compared to 12% of male workers. This gender difference in exposure to the minimum wage increase is consistent with women’s greater concentration in lower-wage jobs.

Indeed, recent evidence on the Spanish reform reveals that the features of the Spanish institutional labor market generate relative wage effects favoring women, especially after the 2019 reform. As discussed above, there was a differential direct wage increase that disproportionately affected females. This finding is consistent with results from Elias and Riudavets-Barcons (2023) documenting that women represented 57.4% of minimum wage earners in the 2018 general workforce. Similarly, Gorjón et al. (2024) find that the 2019 minimum wage increase disproportionately affected women in Spain. Both studies use social security data from the MVCL.

## Data

*Survey on violence against women.* To measure IPV, we rely on the nationally representative cross-sectional Survey on Violence Against Women (SVAW). Although the survey has six waves(1999, 2002, 2006, 2010–11, 2014–15, and 2019), significant methodological changes were introduced in the 2014–15 survey following United Nations recommendations for statistics on violence against women.^5^ Because this methodological change can be considered the beginning of a homogeneous data series, we focus exclusively on the two most recent waves, which were conducted under the Ministry of Equality’s oversight.

As self-reported victimization may underestimate IPV, we follow standard practices in the public health literature and use the 20 objective survey questions, listed in Table 1, that represent different types of psychological, physical and sexual aggression that the woman may have experienced during the last year and where the perpetrator was her partner. The possible answers to the occurrence of each event included “never,” “rarely,” “sometimes,” or “frequently” in the 2015 wave, but were “yes/no” answers in the 2019 wave. Using this information, we follow Brassiolo (2016) and Fernández-Kranz et al. (2024) and define as our main outcome a binary indicator for whether a woman responds “sometimes” or “frequently” to any of the 20 possible violent events in 2015 or “yes” in 2019. We also create binary outcomes to measure the occurrence of different types of violence, namely psychological, physical, and sexual. In each case, the binary outcome indicates whether the woman reports having been victim of any situation or behavior considered by experts to be strong indicators of mistreatment acts under the different types of violence.

**Table 1:** Coding of psychological, physical and sexual violence by partner in past 12 months

*Psychological indicators*	
Verbal abuse and insults from current partner	
Direct verbal threats of physical harm to the respondent	
Intimidation or threats	
Humiliation or degrading treatment	
Threats to other people that are important for the respondent Personal threats (only in 2019)	
*Physical violence indicators*	
Slapping or hitting someone in the face	
Pushing, shoving, or physical aggression	
Punching or hitting with fists	
Kicking or severe physical aggression	
Choking attempts or burning	
Use of or threat with weapons Other type of physical violence (only in 2019)	
*Sexual violence indicators*	
Forced sexual relations through physical coercion	
Sexual relations under fear or threats	
Forced degrading or humiliating sexual practices	
Attempted forced sexual relations	
Sexual relations under influence of alcohol/drugs (only in 2019)	

The reference period is the previous 12 months. While the 2015 wave measured frequency of violence incidents (e.g., Never; Once; Sometimes; Many times); the 2019 wave used a binary (yes/no) approach. From each individual’s responses, binary indicators (0/1) are created where 1 indicates the presence of violence, defined as reporting the behavior “sometimes” or “many times” in 2015 or “yes” in 2019 for any of each categories’ questions

In addition to data on violence, the SVAW collects information about respondents’ and their partners’ socio-demographic characteristics, including age, highest educational degree attained, nationality, marital status, number of children, and employment status. It also includes information on the respondents’ province of residence.^6^

Since the SVAW does not contain wage or earnings data, we use machine learning algorithms trained on a complementary dataset—the *Muestra Continua de Vidas (MCVL)*—which provides detailed wage and employment information. These *MCVL*-trained models are then applied to the socio-demographic data in the SVAW to estimate treatment probabilities. This approach enables us to classify SVAW respondents based on their predicted likelihood of working in a minimum wage job, as outlined in the following subsection.

*Muestra Continua de Vidas Laborales (MCVL) and treatment status.* Using Social Security panel data from the MCVL,^7^ we construct a monthly panel of wage and salary female workers who belong to the general Social Security regime and have worked at least the equivalent to 30 full-time days during a calendar year over the period spanning from January 2018 to December 2018. From this panel, we select the longest episode of employment observed during that period. To maximize representativeness and sample size, our analysis includes both full- and part-time workers, regardless of the number of days worked in each month. For part-time workers, we compute their full-time equivalent monthly wages.^8^

Using this sample of female workers, we trained a model to predict which individuals earned between the old and new minimum wage threshold prior to the 2019 minimum wage increase, based on observable characteristics shared across both datasets (e.g., age, education, children at home, immigrant status, and province). Specifically, we constructed a binary indicator to identify whether a woman’s earnings in 2018—before the minimum wage increase—were between the old and the new minimum wage threshold. Women whose pre-2019 monthly wages fall within the range between 858.55 and 1,050 euros (for 12 payments per year) are assumed to represent the treated group, as their earnings were directly impacted by the minimum wage reform. Appendix Table 7 shows the socio-demographic characteristics of female workers in the MCVL working in a minimum wage job versus those who do not. Women working a minimum wage job are younger, less educated, more likely to be immigrant, and have more children.

To estimate treatment status based on socio-demographic characteristics, we employ machine learning techniques (see Autor et al. 2023; Cengiz et al. 2022a, b). More specifically, we use the LASSO (Least Absolute Shrinkage and Selection Operator) method to predict treatment status based on observable characteristics, with the resulting model serving as a classification tool to stratify individuals into treatment and comparison groups.^9^ In our main specification, we interact all observed female worker characteristics (age, five age categories, four education levels, immigrant status, having children, and province) to capture nonlinear relationships. We first train the model on Social Security data from the MCVL, where actual wages are observed. This data-driven method of variable selection is particularly valuable in our context, as we must identify treated individuals without direct wage information in the SVAW data. Importantly, it avoids arbitrary decisions about which characteristics are most predictive of low-wage status.

The MCVL-trained model^10^ is then applied to generate predicted probabilities of treatment (i.e., being affected by the minimum wage reform) for individuals in our main dataset, SVAW, which focuses on IPV, yet lacks wage information. Since the model relies on demographic and educational variables rather than employment variables, it can estimate the likelihood of an individual being affected by the policy even if she is currently unemployed or inactive. This allows us to examine the policy’s effects not only on employed individuals but also on those who are currently not employed, that is the intention-to-treatment effects.

This data-driven classification, while a substantial improvement over standard education-based proxies, may introduce some measurement error, which we assess in detail in Sect. 5 through a range of robustness checks.

We define the treatment group as SVAW respondents with a predicted probability of being affected by the minimum wage increase at or above the median and the comparison group as those with a predicted probability below the median.^11^ As emphasized by Cengiz et al. (2022a, b), this classification strategy is intended to group individuals, rather than to draw inferences from individual covariates. Indeed, our final model generate “good” predictions. For instance, when we use the median to classify individuals into the treated and comparison group, the model achieves a precision rate of 73%. This means that 73% of individuals classified as “treated” (likely affected by the minimum wage increase) are correctly identified as minimum wage workers according to Social Security records. If instead of the median, we classify treated individuals with those in the higher 60th percentile and comparison individuals with those in the lower 40th percentile, our precisions rate increases to 79%. In the robustness section, we used this alternative classification, and show results consistent with our main findings. We prefer to use the median probability as dividing threshold to ensure the largest possible sample size for subgroup analysis. Finally, it is important to note that this approach to proxy low-income workers reflects a substantial improvement over the standard practice in the literature, which often uses proxies such as education level attainment (e.g., “no college degree”)—see Hoynes et al. (2015) and related literature.

*Sample restriction and descriptive statistics*. We restrict the sample to women 18–65 years old, eliminating women over the retirement age. We also restrict the sample to women who have a current partner. This leaves us with a sample of 11,360 women, 5,343 of which are from the 2019 wave. Table 2 presents descriptive statistics for women based on their predicted probability of working in a minimum wage job. As expected, there are significant socio-demographic differences across the two groups. Women with a high predicted probability of working in a minimum wage job are younger, less educated, and live in smaller municipalities than those with a low predicted probability. Furthermore, they are more likely to be immigrants and religious than those with a low predicted. Their partners are also younger and less educated than those of women with a low predicted probability of working at a minimum wage job.

**Table 2:** Main sample characteristics of females (Survey IPV 2015–2019)

		(1) Treatment mean/sd	(2) Control mean/sd	(3) Difference mean/*p* value
Female characteristics	*Educational Attainment Level*			
	Primary or less	0.083	0.027	0.056^***^
		(0.282)	(0.116)	0.000
	Secondary	0.769	0.392	0.378^***^
		(0.427)	(0.489)	0.000
	High school	0.109	0.123	− 0.013^*^
		(0.296)	(0.348)	0.000
	University	0.039	0.459	− 0.420^***^
		(0.230)	(0.498)	0.000
	*Municipality size*			
	Small	0.496	0.449	0.047^***^
		(0.500)	(0.498)	0.000
	Medium	0.381	0.368	0.013^***^
		(0.487)	(0.480)	0.003
	Large	0.123	0.183	− 0.060^***^
		(0.327)	(0.389)	0.000
	*Religion*			
	Catholic	0.644	0.681	0.037^***^
		(0.409)	(0.461)	0.000
	No religion	0.356	0.319	− 0.045^***^
		(0.409)	(0.461)	0.000
	Age (Years)	36.90	48.78	− 10.191^***^
		(11.677)	(9.878)	0.000
	Immigrant	0.249	0.042	0.207^***^
		(0.421)	(0.252)	0.000
	Has children	0.726	0.812	− 0.086^***^
		(0.456)	(0.388)	0.000
Partners Characteristics	*Age of the partner*			
	Age (Years)	40.23	50.7	− 10.7^***^
		(.146)	(.139)	0.000
	Less than 35	0.106	0.003	0.103^***^
		(0.308)	(0.053)	0.000
	35–44	0.263	0.086	0.176^***^
		(0.440)	(0.280)	0.000
	45–54	0.277	0.259	0.019^**^
		(0.448)	(0.438)	0.029
	55–64	0.206	0.308	− 0.102^***^
		(0.404)	(0.462)	0.000
	*Partner education*			
	Primary or less	0.125	0.118	0.007
		(0.359)	(0.445)	0.000
	Secondary	0.532	0.496	0.036^***^
		(0.336)	(0.296)	0.000
	High school	0.133	0.130	0.003
		(0.485)	(0.424)	0.000
	University	0.209	0.256	− 0.046^**^
		(0.338)	(0.339)	0.853
	Immigrant Partner	0.149	0.035	0.207^***^
		(0.356)	(0.035)	0.000
	Observations	5,439	5,921	11,360

Standard deviations in parentheses. Differences computed as Treatment—Control. *p < 0.10, **p < 0.05, ***p < 0.01

Before the minimum wage increase, 6.73% of women reported having experienced some form of physical, sexual or psychological abuse by an intimate partner in the last 12 months. The vast majority (6.46%) reported being victims of psychological abuse. Additionally, 1.16% and 0.91% reported being victims of physical and sexual violence, respectively.

As documented in the literature, young age, low education, and poverty are well known risk factors for IPV (WHO 2010). Payne (2020) also finds higher IPV prevalence rates among immigrant women. Consistent with this, Table 3 documents a higher prevalence of physical and sexual IPV among women in the Treatment group (i.e., those with a higher predicted probability of working in a minimum wage job) than those in the comparison group prior to the reform.

**Table 3:** Measures of intimate partner violence: descriptive statistics (Survey IPV 2015–2019)

	2015			2019		
	Treatment	Control	Diff	Treatment	Control	Diff
IPV all	0.070	0.065	0.005	0.067	0.045	0.022^***^
	(0.255)	(0.246)	[0.432]	(0.251)	(0.208)	[0.001]
IPV Psychological	0.066	0.063	0.003	0.065	0.043	0.022^***^
	(0.249)	(0.243)	[0.641]	(0.246)	(0.202)	[0.000]
IPV Physical	0.015	0.009	0.006^**^	0.011	0.006	0.006^**^
	(0.120)	(0.093)	[0.034]	(0.104)	(0.076)	[0.050]
IPV Sexual	0.012	0.007	0.005^*^	0.013	0.008	0.005^*^
	(0.107)	(0.082)	[0.053]	(0.114)	(0.088)	[0.001]
Observations	2,932	3,085	6,017	2,507	2,836	5,343

Standard deviations in parentheses. Differences computed as Treatment—Control. *p < 0.10, **p < 0.05, ***p < 0.01

Appendix Table 8 presents these same characteristics separately by survey wave (2015 and 2019), confirming that the observable differences between treatment and control groups remain relatively stable across both periods, supporting the validity of our difference-in-differences design.

## Empirical strategy

To estimate the impact of the minimum wage reform on IPV, we first estimate a Differences-in-difference model as stated below:

$$ {\mathrm{IPV}}_{it} = \alpha + \beta_{0} *{\mathrm{Treated}}_{i} + \beta_{1} *{\mathrm{Post}}_{t} + \beta_{3} *{\mathrm{Post}}_{t} *{\mathrm{Treated}}_{i} + \gamma X_{it} + \delta_{p} + \varepsilon_{it} $$

where ${\mathrm{IPV}}_{it}$ is a measure of the IPV experienced by individual *i*, in year *t*; ${\mathrm{Treated}}_{i}$ is a binary variable that takes value equal to one if the individual is in the treatment group, and zero otherwise. The treatment group is composed of individuals with an above-the-median predicted probability of being affected by the minimum wage increase prior to the reform. ${\mathrm{Post}}_{t}$ takes value equal to one if the survey is completed in 2019, and zero otherwise.^12^$X_{it}$ is a set of covariates such as age, highest educational attainment dummies (primary or less, secondary, high school, and university), municipality size (small, medium, large), religious affiliation (Catholic, no religion), immigrant status, presence of children in the household, partner’s age, partner’s immigrant status, and partner’s educational attainment (primary or less, secondary, high school, university), and province fixed effects ($\delta_{{\mathrm{p}}}$). Our coefficient of interest is $\beta_{3}$. It estimates the impact of the minimum wage increase on individuals’ victimization by their intimate partner. In order to understand the mechanisms driving our results, the left-hand-side variable will also include both the woman’s employment and unemployment status as well as that of her partner. We also estimate the effect of the reform on changing marital status to estimate potential compositional effects of the reform.

*Inverse probability weighted robust DiD.* Because eligible workers are not directly observed as working at the minimum wage, but instead predicted using a LASSO-trained model from administrative data, this introduces strong imbalance between treated and comparison groups as individuals are selected from different parts of the predicted distribution. To reduce these imbalances, we implement a **doubly robust difference-in-differences (DiD)** estimator using **inverse probability weighting (IPW)**, following Sant’Anna and Zhao (2020). This approach combines a propensity score model and an outcome regression, both based on the same set of covariates, and ensures consistent estimation of the average treatment effect on the treated (ATT) if either model is correctly specified. To preserve estimator robustness and mitigate numerical instability due to limited common support, we apply trimming at the 5st and 95th percentiles. Our procedure consists of three steps: (1) estimate propensity scores via logistic regression using socio-demographic characteristics of women and their partners—age, education (four categories), municipal size, religious affiliation, immigrant status, presence of children, and province fixed effects; (2) construct IPW weights as 1/p for treated and 1/(1–*p*) for controls; and (3) estimate a weighted DiD regression that includes the same covariates used in the propensity score model to achieve double robustness and control for unobserved time-invariant heterogeneity.

**Fig. 3:**
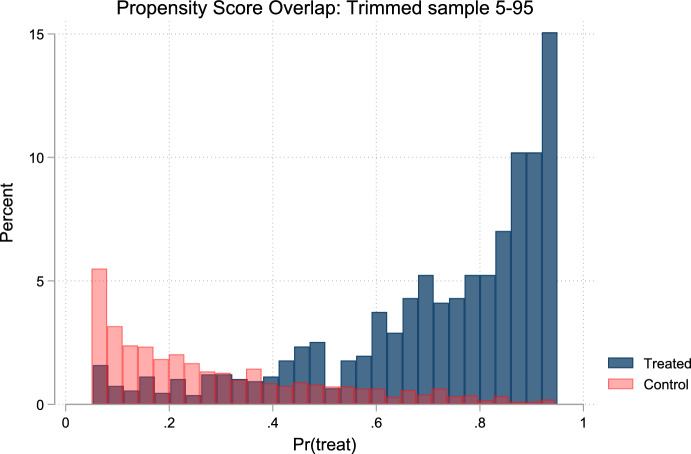
Common Support for the Propensity Score to Work in a Minimum Wage Job (Treated versus comparison groups, SVAW) Notes: Histogram shows the distribution of predicted probabilities of working in a minimum wage job after trimming at the 5th and 95th percentiles. Dark blue bars represent the treated group (above median predicted probability) and light pink bars represent the control group (below-median predicted probability). The clear separation between distributions confirms successful identification of distinct treatment and comparison groups. Propensity scores estimated using LASSO logistic regression on socioeconomic characteristics from the SVAW 2015–2019

To assess the validity of our inverse probability weighting (IPW) approach within the DiD framework, we evaluate the distribution of propensity scores and the resulting covariates balance. Figure 3 illustrates the distribution of estimated propensity scores—based on socio-demographic characteristics of women and their partners from the SVAW 2015–2019. The figure shows substantial overlap in the distribution of propensity scores between treated and comparison groups, confirming adequate common support for the IPW estimation. This overlap ensures that treated and comparison units can be meaningfully compared across the range of predicted treatment probabilities.

*Covariate balance assessment* Appendix Table 9 reports covariates balance diagnostics before and after weighting for all variables included in the propensity score model. The IPW procedure substantially improves balance across most covariates. For instance, we observe reductions in standardized mean differences of 33% for age and 30% for immigrant status, along with notable gains in educational attainment. Variables that were already balanced in the unweighted sample—such as partner’s education—remain stable after weighting. These results confirm that our weighting procedure effectively mitigates observable selection bias, reinforcing the credibility of our identification strategy.

## Results on IPV

*Impact on IPV* Panel A in Table 4 presents estimates of $\hat{\beta }_{3}$, our coefficient of interest, measuring the effect of the minimum wage increase on IPV using both the regular DiD model and robust DID-IPW model.^13^ Columns (1) and (3) show the estimates from models without covariates. All other columns show estimates from models with all covariates. All $\hat{\beta }_{3}$ estimates are positive and statistically significant at 10% level or lower, indicating that the minimum wage hike increased the incidence of IPV in Spain by a range between 25 and 40%. Our preferred model, the DID-IPW model controlling for covariates (shown in column 4), reveals that the minimum wage reform increased the incidence of IPV by 2.7 percentage points. The magnitude of this effect is economically significant, representing an increase of 40% relative to the pre-reform control group IPV rate of 6.73%. Panels B–C present separate estimates for the different types of violence: psychological (Panel B), physical (Panel C), and sexual (Panel D). Focusing on our preferred specification, shown in column 4, we observe that most of the increase in violence is driven by psychological violence (a 2.7 percentage points or 42% increase).

**Table 4:** Effect of Minimum Wage on Intimate Partner Violence and Having a Partner: By Violence Type

	Model 1: DID		Model 2: DID-IPW		Model 2: DID-IPW	
					40 versus 60 percentile	Continuous treatment (MW exposure)
	(1)	(2)	(3)	(4)	(5)	(6)
*Panel A. Overall IPV*						
Treated × Post	0.017**	0.019**	0.023*	0.027**	0.021**	0.065**
	(0.01)	(0.01)	(0.01)	(0.01)	(0.01)	(0.03)
IPV (%)	6.73	6.73	6.73	6.73	6.67	6.73
*Panel B. Psychological IPV*						
Treated × Post	0.019**	0.020**	0.024*	0.027**	0.022**	0.067**
	(0.01)	(0.01)	(0.01)	(0.01)	(0.01)	(0.03)
IPV (%)	6.47	6.47	6.47	6.47	6.41	6.47
*Panel C. Physical IPV*						
Treated × Post	− 0.000	− 0.000	0.002	0.001	0.001	− 0.004
	(0.00)	(0.00)	(0.00)	(0.00)	(0.00)	(0.01)
IPV (%)	1.16	1.16	1.16	1.16	1.17	1.16
*Panel D. Sexual IPV*						
Treated × Post	0.001	0.002	0.002	0.002	0.001	0.008
	(0.00)	(0.00)	(0.00)	(0.00)	(0.00)	(0.01)
IPV (%)	0.91	0.91	0.91	0.91	0.89	0.91
*Panel E. Having a partner*						
Treated × Post	− 0.005	0.001	− 0.005	− 0.006	0.007	− 0.023
	(0.02)	(0.01)	(0.02)	(0.02)	(0.02)	(0.04)
Having a partner (%)	77.99%	77.99%	77.99%	77.99%	77.37%	77.99%
Covariates	(No)	(Yes)	(No)	(Yes)	(Yes)	(Yes)

Robust standard errors clustered at provincial level in parentheses. Model 1 DID refers to Difference-in-Differences, and Model 2 DID-IPW refers to Double Robust Difference-in-Differences using Inverse Probability Weighting. Outcomes means are for the comparison group pre-reform. Covariates included in all models except the one corresponding having a partner where covariates related to the partner are not included: Age, Education, Immigrant, having a child, Municipality Size, Region of residence, Capital Municipality, age of the partner, education of the partner and immigrant partner. These covariates are defined in Table 2 above. Sample size is 11,360 women except in Panel E where it is 14,900 and in Column 5 where it is 10,181. *p < 0.10, **p < 0.05, ***p < 0.01

While the magnitude of our estimated effects may seem large, our findings are well within the range of those documented in prior research for Spain. For example, Arenas-Arroyo et al. (2021) find that psychological IPV under forced cohabitation during COVID-19 in Spain increased by 30% relative to pre-COVID-19 levels with no effect on physical violence. In a different study, Fernández-Kranz et al (2024) estimate that the Spanish joint custody laws reduced the incidence of IPV by 45% as fathers’ expected utility upon divorce increased and their bargaining power within the household improved, subsequently reducing their use of violence to prevent a divorce.

It is noteworthy that adding covariates to either model slightly increases the estimated effects on IPV and psychological IPV (by no more than 0.4 percentage points), while leaving the estimates for sexual and physical IPV unchanged. Under the assumption that unobserved heterogeneity would bias the estimates in the same direction as observed heterogeneity, this pattern suggests that our estimates may understate the true increase in psychological violence. A similar pattern is observed when moving from our DiD estimates to double robust DiD using inverse probability weighting. While various societal changes occurred between 2015 and 2019—including the rise of Vox, the aftermath of high-profile cases like La Manada, the #MeToo movement, and housing price increases—these factors would only confound our estimates if they differentially affected treatment and control groups in ways correlated with minimum wage exposure, conditional on our controls including province fixed effects, education, age and, municipality size. We have no theoretical or empirical reason to expect such differential exposure, especially given that our estimates with and without covariates or moving from DiD estimates to double robust DiD using inverse probability weighting suggest that if anything confounding biases would underestimate the true effect on IPV.

**Fig. 4:**
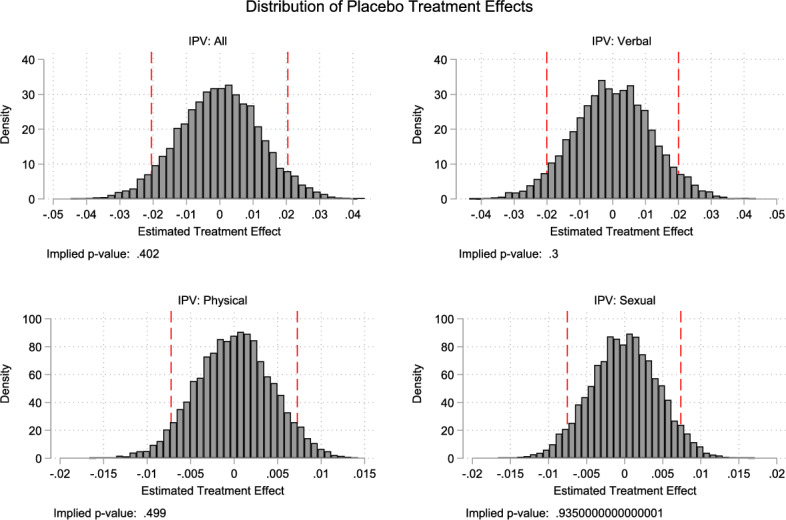
Distribution of Placebo treatment Effects for each type of IPV *Notes:* we conduct 10,000 iterations of the following procedure within our comparison group sample. First, we randomly assign placebo treatments to create artificial treatment and comparison groups. In each iteration, we implement the full robust estimation procedure, which involves: (i) estimating the propensity score model for the placebo treatment and (ii) using these components to construct the DID robust estimate. This approach allows us to generate a distribution of placebo effects that maintains the essential features of our primary estimation strategy. These figures present the distribution of the placebo estimates for the overall IPV outcome and each of the three IPV categories: psychological, physical, and sexual. The implied p values cannot reject the null hypothesis of no effect, suggesting that our main findings are not random findings

*Placebo tests* As we cannot test the parallel trend assumption directly, we conduct 10,000 placebo iterations within the comparison group. In each iteration, we randomly assign treatment status and estimate the full DID-IPW model. The resulting distribution of placebo effects—shown in Fig. 4 for overall and type-specific IPV outcomes—centers around zero and lacks statistical significance, reinforcing that our main findings are unlikely to be due to chance.

*Additional robustness checks* To address concerns about potential measurement error in our treatment classification, we implemented a more conservative classification that restricts the treatment and comparison groups to individuals with predicted probabilities above the 60th percentile and below the 40th percentile, respectively. This approach creates a clearer separation between treated and comparison units by excluding individuals with ambiguous treatment status, thus reducing the potential for misclassification. As explained in Sect. 3 above, with this specification, the precision rate rises to 79%. The results from this more conservative specification (shown in column 5 in Table 4) show similar effect sizes compared to our main estimates with maintained statistical significance. This pattern suggests that our main findings are unlikely to be driven by measurement error in the treatment assignment.

Concerns that classifying treatment based on a binary threshold may mask important variation in the intensity of exposure to the minimum wage reform are addressed column 6 in Table 4, where we estimate a specification using a continuous treatment variable based on the predicted probability of being a minimum wage worker (treatment intensity). This approach allows us to capture more granular variation in treatment intensity, including differences between individuals with marginal versus substantial exposure to the reform. This concordance across approaches—which impose fundamentally different functional form assumptions—confirms that our findings are robust to treatment measurement choices and are not artifacts of arbitrary classification thresholds.

Concerns that our results are driven by compositional changes caused by the reform are addressed in Panel E in Table 4. In particular, we investigate whether the 2019 minimum wage reform had any effect on civil status—specifically, whether it influenced the likelihood of women having a partner (including cohabiting partners and husbands) at the time of the interview. We estimated the impact of the reform on the probability of having a partner or husband using the full sample of women aged 18–65 years old in the *Survey on Violence Against Women*. Estimates show that the reform had no statistically significant effect on the likelihood of having a partner or husband. This finding provides reassurance that our restriction to partnered women does not introduce selection bias due to differential partnership status induced by the reform.

Finally, we also verified that our psychological violence results are not driven by the addition of the “personal threats” question in the 2019 survey wave. We find that no respondents in 2019 report psychological violence exclusively through this new item; all women classified as experiencing psychological violence also reported at least one form of abuse measured in both survey waves. Restricting our indicator to only the common questions yields substantively identical results.

## Underlying mechanisms

Theoretical frameworks—such as household bargaining, the backlash model, or the instrumental model—typically rely on changes in *relative* earnings between partners, yet our minimum wage reform applies broadly to both genders. However, as explained in Sect. 2, the Spanish institutional labor market is such that recent evidence by Elias and Riudavets-Barcons (2023) and Gorjón et al. (2024) support gender-differentiated effects of the 2019 minimum wage increase.

To fully understand the mechanisms driving this reform’s increase in partner psychological violence, we must evaluate the impact of the reform on both partners’ labor market. If the minimum wage reform increases female labor market attachment or reduces female unemployment relative to that of their male partner, given that the reform increased psychological IPV, this would be supportive of the male backlash or instrumental theoretical frameworks. These models would also hold if the increase in IPV comes with no relative effect on women’s labor force status, since the reform implies an increase in earnings for those who work minimum wage jobs even if there is no employment effect. Alternatively, if the minimum wage reform reduces female labor market attachment or increases female unemployment relative to that of their partner, since it increases IPV, this would be suggestive that the economic bargaining power theory is at works.

To better understand intra-household employment dynamics, Table 5 presents estimates using as left-hand-side variables: female employment and unemployment (Panels A and B); their partners’ employment and unemployment (Panels C and D); and both partners’ employment (Panel E). It is important to remind the reader that this paper's analysis is limited to married or cohabitating couples. Consequently, our employment estimates of the 2019 minimum wage increase may differ from those of Barceló et al. (2021), Elias and Riudavets-Barcons (2023), Gorjón et al. (2024), and Casanova et al. (2025), as those studies analyze representative samples of the Spanish population that include single, divorced, separated, and non-cohabitating individuals.

**Table 5:** Effect of minimum wage: impact on labor market outcomes within the household

	Model 1: DID		Model 2: DID-IPW		Model 2: DID-IPW	
					40 versus 60 percentile	Continuous treatment (MW exposure)
	(1)	(2)	(3)	(4)	(5)	(6)
*Panel A. Woman employed (1* = *Yes)*						
Treated × Post	− 0.050**	− 0.043**	− 0.019	− 0.031	− 0.037*	− 0.107
	(0.02)	(0.02)	(0.03)	(0.02)	(0.02)	(0.06)
Mean	51.64	51.64	51.64	51.64	51.76	51.65
*Panel B. Woman unemployed (1* = *Yes)*						
Treated × Post	− 0.003	0.009	0.020	0.020	0.026	0.030
	(0.02)	(0.02)	(0.02)	(0.02)	(0.02)	(0.05)
Mean	25.16	25.16	25.16	25.16	25.48	25.15
*Panel C. Partner employed (1* = *Yes)*						
Treated × Post	0.008	0.028**	0.046*	0.037**	0.025	0.030
	(0.02)	(0.01)	(0.02)	(0.01)	(0.02)	(0.03)
Mean	70.88	70.88	70.88	70.88	71.13	70.88
*Panel D. Partner unemployed (1* = *Yes)*						
Treated × Post	− 0.036***	− 0.036***	− 0.033*	− 0.040***	− 0.050***	− 0.054*
	(0.01)	(0.01)	(0.02)	(0.01)	(0.02)	(0.03)
Mean	13.56	13.56	13.56	13.56	13.61	13.56
*Panel E. Both partners employed (1* = *Yes)*						
Treated × Post	− 0.033	− 0.019	0.002	− 0.005	− 0.004	− 0.037
	(0.02)	(0.02)	(0.03)	(0.03)	(0.03)	(0.07)
Mean	41.30	41.30	41.30	41.30	41.60	41.30
Covariates	No	Yes	No	Yes	Yes	Yes

In this analysis, we use a binary variable for paid employment that takes the value 1 when a woman has a remunerated job and 0 otherwise (which includes unemployment and inactivity). This variable equals 1 for women who report works or works or habitually collaborates in the family business. The variable equals 0 for all other categories, including unemployed and has worked before, unemployed and seeking first job, retired or pensioner, student, unpaid domestic work, and other situation. We also construct a separate unemployment variable that takes the value 1 when a woman is unemployed and 0 otherwise, which includes only those women reporting being employed

Robust standard errors clustered at provincial level in parentheses. Model 1 DID refer to Difference-in-Differences, and Model 2 DID-IPW refers to Double Robust Difference-in-Differences using Inverse Probability Weighting. Covariates used are the same as those in Table 3. Sample size is 11,360 women except in Column 5 where it is 10,181. *p < 0.10, **p < 0.05, ***p < 0.01

All estimates in Panel A in Table 5 are negative, and three of six are statistically significant at the 10 percent level or lower. Our preferred specification reveals that women’s employment decreased by 3 percentage points, or 6% of the pre-reform mean of 52%. While this estimate lacks precision, the one in column 5, where we define treated and comparison groups based on the 60th and 40th percentiles, is a tad larger and statistically significant at the 10% level. Estimates on the likelihood of being unemployed (in Panel B) are all positive, yet not statistically significantly different from zero in any specification.

Interestingly, Panels C and D reveal that the partners of the women in our sample increased their employment by 3.7 percentage points or 5% of the pre-reform mean of 71%. Similarly, their unemployment decreased by 29% (a 4 percentage points from a pre-reform level of 13.6%). Both estimates are statistically significant at the 5% level or lower. Taken together, estimates in Table 5 suggest that the policy triggered complex household labor supply responses, potentially reflecting substitution effects between spouses. As shown in Panel E, the gains in male employment were sufficient to fully offset the losses in female employment, resulting in no net change in total household employment. These dynamics may contribute to increased IPV through reduced female bargaining power within the household.

Our estimates are unlikely to be confounded by broader societal changes between 2014 and 2019—such as the rise of Vox, the *Me Too* movement, or the aftermath of *La Manada*—because our DiD strategy differences out national-level shocks affecting all women and men. Moreover, if cultural shifts were driving the results, we would expect uniform change in rather than the differential effects we observe, which are consistent with economic disempowerment.

At first glance, our estimated 6% reduction in female employment may appear larger than what has been reported in some of the recent studies analyzing the 2019 minimum wage reform in Spain (Gorjón et al. 2024; Elias and Riudavets-Barcons 2023). However, it is crucial to recognize that our analysis focuses on women living with a partner, who tend to be secondary earners within their households.^14^ This group is likely to exhibit a more elastic labor supply response to wage changes, which is consistent with the employment reduction we observe. This mechanism differs from those captured in studies analyzing broader populations. Our findings are therefore not inconsistent with the existing literature but rather complement it by highlighting heterogeneity in employment responses to minimum wage reforms—particularly among partnered women whose labor force participation is more sensitive to household income dynamics.

Moreover, other recent studies using administrative or firm-level data also document non-negligible negative employment effects. For example, Casanova et al. (2025) finds that the 22% minimum wage increase led to a 5 percentage point decline in employment growth in fully affected firms, with stronger impacts in small establishments and significant increases in worker turnover. Similarly, Barceló et al. (2021) use the MCVL and find a net employment aggregate loss of between 6 and 11% for workers directly affected by the 2019 reform. Taken together, our findings are well within the range of credible evidence and highlight an important mechanism—labor force withdrawal by secondary earners. They also are consistent with an interpretation that partnered women—especially those with less attachment to the labor market—respond to income shocks differently than primary earners or the average worker.

*Heterogeneity analysis* Table 6 display subgroup analysis by women’s education and age, and by differences between partners’ education and age. Table 6 reveals that the increase in psychological violence is driven by households where the woman does not have a college degree (column 1) or the man is five years senior to her (column 4). For women without a college degree, the reform reduces their employment by 12% from a pre-reform level of 46.2%, without impacting their partners’ employment (shown in Table 6). As women with less than a college degree reduce their employment relative to their partners after the reform, their bargaining power within the household decreases, and psychological violence increases. Based on Table 2, 94% of women in our sample did not earn a college degree.

**Table 6:** Heterogeneity analysis: DID-IPW estimates

	Education (1)	Ed. differences (2)	Age (3)	Age differences (4)
*A*				
PANEL A (Any Type IPV)				
Treatment × Post × Group 1	0.046	0.018	0.019	0.065***†
	(0.03)	(0.02)	(0.01)	(0.02)
Treatment × Post × Group 2	0.035**	0.028	0.032	0.017†
	(0.01)	(0.02)	(0.03)	(0.02)
Mean G1 IPV (%)	5.09	4.95	6.90	8.56
Mean G2 IPV (%)	7.24	6.98	6.02	6.31
PANEL B (Psychological)				
Treatment × Post × Group 1	0.042	0.019	0.020	0.071***††
	(0.03)	(0.03)	(0.01)	(0.02)
Treatment × Post × Group 2	0.035**	0.029	0.028	0.015††
	(0.02)	(0.02)	(0.03)	(0.02)
Mean G1 IPV (%)	5.02	4.68	6.65	8.02
Mean G2 IPV (%)	6.92	6.72	5.67	6.11
PANEL C (Physical)				
Treatment × Post × Group 1	− 0.018†	− 0.007	0.004†	0.007
	(0.01)	(0.01)	(0.01)	(0.01)
Treatment × Post × Group 2	0.006†	0.003	− 0.010*†	0.000
	(0.01)	(0.00)	(0.01)	(0.00)
Mean G1 IPV (%)	0.35	0.53	1.07	2.05
Mean G2 IPV (%)	1.42	1.25	1.55	0.96
PANEL D (Sexual)				
Treatment × Post × Group 1	− 0.002	− 0.003	0.000††	0.006
	(0.01)	(0.01)	(0.00)	(0.01)
Treatment × Post × Group 2	0.005	0.004	0.015**††	0.002
	(0.00)	(0.00)	(0.01)	(0.00)
Mean G1 IPV (%)	0.42	0.80	0.91	1.60
Mean G2 IPV (%)	1.07	0.93	0.95	0.76
*B*				
PANEL A (Woman employment)				
Treatment × Post × Group 1	0.025	− 0.093*	− 0.021	0.043
	(0.11)	(0.05)	(0.03)	(0.07)
Treatment × Post × Group 2	− 0.054*	− 0.028	0.057	− 0.032
	(0.03)	(0.03)	(0.06)	(0.03)
Mean G1 employment (%)	69.09	46.79	53.73	48.40
Mean G2 employment (%)	46.20	52.34	42.99	52.40
PANEL B (Woman Unemployment)				
Treatment × Post × Group 1	− 0.044	0.102**	0.038	− 0.049††
	(0.12)	(0.04)	(0.03)	(0.04)
Treatment × Post × Group 2	0.017	− 0.000	− 0.063	0.036††
	(0.03)	(0.04)	(0.07)	(0.03)
Mean G1 employment (%)	19.40	24.87	23.51	26.83
Mean G2 employment (%)	26.94	25.19	31.99	24.76
PANEL C (Partner employment)				
Treatment × Post × Group 1	0.223**	0.022	0.039*	0.054
	(0.09)	(0.04)	(0.02)	(0.06)
Treatment × Post × Group 2	0.022	0.033	0.028	0.055*
	(0.02)	(0.02)	(0.05)	(0.03)
Mean G1 employment (%)	83.81	74.87	71.14	60.96
Mean G2 employment (%)	66.84	70.32	69.82	73.16
PANEL D (Partner Unemployment)				
Treatment × Post × Group 1	− 0.283**	− 0.036†††	− 0.034*†††	− 0.015
	(0.11)	(0.04)	(0.02)	(0.03)
Treatment × Post × Group 2	− 0.010	− 0.037**†††	− 0.040†††	− 0.044**
	(0.02)	(0.02)	(0.05)	(0.02)
Mean G1 unemployment (%)	8.23	10.03	12.36	14.88
Mean G2 unemployment (%)	15.23	14.06	18.57	13.26
PANEL E (Both partners employed)				
Treatment × Post × Group 1	0.168†††	− 0.062	− 0.003	0.035
	(0.10)	(0.06)	(0.03)	(0.06)
Treatment × Post × Group 2	− 0.038†††	0.003	0.090	− 0.013
	(0.02)	(0.03)	(0.07)	(0.02)
Mean G1 employment (%)	60.64	38.90	42.91	33.33
Mean G2 employment (%)	35.25	41.64	34.57	43.13

Robust standard errors clustered at provincial level in parentheses: Outcomes Column (1) Education: Group 1 (University degree); Column (2) Educational differences: Group 1 (male higher education than women); Column (3) Age: Group 1 (> 30), Column (4) Age differences: Group 1 (> 5 years male older). Covariates used are the same as those in Table 3. All models refer to our reference model 2 DRDID IPW. Sample size is 11,360 women

^*^p < 0.10, **p < 0.05, ***p < 0.01

^†^*p* < 0.10, ††*p* < 0.05, †††*p* < 0.01 *P* values for test of coefficient from Group 1 equal to coefficient from Group 2

In contrast, for women with an older partner (representing 20.6% of our sample), although we lack precision in the labor market estimates, the coefficients point to both partners increasing employment. To the extent women tend to be more minimum wage workers than men, this would imply that the violence is the result of women’s relative gain in earnings, which would be suggestive of a backlash model or instrumental use of violence by the male partner. A similar conclusion could be draw for younger women (shown in Column 3), for whom we observe a substitution from physical to sexual violence after the reform with higher labor market involvement from both partners (albeit not statistically significant). For both subgroups, we can rule out violence driven by an increase in financial stress as both partners’ employment increases.

Finally, there is no evidence that the reform impacted the risk of victimization among couples where he has a higher educational attainment than her (Column 2), even though women lower their labor market attachment after the reform.

## Conclusion

Using data from 2014 to 2015 and 2019 *Survey of Violence Against Women*, we estimate a significant increase in the Spanish minimum wage in 2019 on intimate partner violence. We do not find that the reform, which increased the minimum wage by 22% in Spain, a country known for a higher concentration of women in minimum wage jobs. Furthermore, we find that the minimum wage led to a 40% *increase* in intimate partner victimization, mostly driven by psychological violence. Subgroup analysis reveals that the effect is primarily driven by women without a college degree (which is the majority of our sample) and those with partners who are at least five years older. For women without a college degree, labor market analysis reveals that the reform led to a substitution away from female employment toward that of her partner, reducing women’s bargaining power within the household.

For women with a partner at least five years older, the increase in violence is likely the result of gender role conflict, or instrumental use of violence, as violence increases with no labor market displacement. In such case, the policy recommendations would be to accompany the minimum wage reform with norm-evolving policies that would directly challenge societies’ hegemonic masculine norms. Such policies include public health awareness campaigns and as well as any policy or program that would propagate alternative forms of masculinity in society (e.g., by incorporating them in teaching curricula as early as in primary education).

Policymakers should seek to understand—and address—the factors driving the reduction in female employment following the minimum wage increase, as economic empowerment (or a reduction of it due to women’s lower labor market attachment) may underlie the observed rise in violence. Most importantly, our findings are not to be read as a warning against increasing earnings of low-income workers. Instead, they underscore the nuances and complexities of labor market policies on intimate partner relationships and highlight the need for specific policies raising public awareness on the red flags and dangers of gender-based and domestic violence, as well as the need for more victims’ services targeted those groups most at risk of victimization.

This analysis is subject to several limitations that merit discussion. First, due to data constraints, we do not observe actual wages in the *Survey on Violence Against Women* (SVAW), which prevents us from directly measuring income changes following the minimum wage reform. To address this, we constructed a predictive model of minimum wage exposure using detailed labor market data from the 2018 *Muestra Continua de Vidas Laborales* (MCVL), which we then applied to the SVAW sample based on shared socio-demographic characteristics. While this method allows us to approximate treatment status, it may introduce some measurement error. To assess the robustness of our findings, we conducted several complementary analyses. These include varying the thresholds used to define treatment intensity, testing alternative specifications of the treatment probability model, and performing placebo tests. Additionally, we examined whether the reform led to significant changes in sample composition—such as shifts in partnership status—that could bias our estimates. Across these checks, our results remain stable, supporting the validity of our main conclusions while also highlighting the empirical challenges inherent in studying intimate partner violence using available data sources.

Finally, while data limitations prevent us from estimating event-study models or observing longer pre-treatment trends, it is important to underscore that we adopt a robust empirical strategy to mitigate these concerns. Specifically, we implemented an inverse probability weighted difference-in-differences (DID-IPW) design that balances observed characteristics across treatment and comparison groups while controlling for unobserved time-invariant heterogeneity. This approach, together with extensive robustness checks, placebo tests, and heterogeneity analyses, strengthens the credibility of our findings. Although we cannot entirely eliminate concerns about omitted variable bias, the consistency of results across specifications and subgroups suggests that our estimates capture meaningful causal effects. Within the constraints of the available data, our approach represents an improvement over certain standard methods used in the literature and contributes valuable evidence on the broader consequences of minimum wage policies.

**Table 7:** Main sample characteristics of females (*MCVL,* 2018)

	(1)	(2)	(3)
	MW Female workers	Female workers above MW	Difference
*Educational Attainment Level*			
Primary or less	0.308	0.029	0.279***
	(0.462)	(0.168)	0.000
Secondary	0.471	0.180	0.291***
	(0.499)	(0.385)	0.000
High School	0.151	0.236	0.085***
	(0.358)	(0.424)	0.000
University	0.070	0.555	− 0.485***
	(0.255)	(0.497)	0.000
Has children	0.391	0.388	0.003**
	(0.488)	(0.487)	0.037
Spanish nationality	0.830	0.996	− 0.166***
	(0.375)	(0.062)	0.000
*Age*			
Age Groups			
Age < 34	0.304	0.166	0.137***
	(0.460)	(0.372)	0.000
Age 35–44	0.278	0.340	− 0.062***
	(0.448)	(0.474)	0.000
Age 45–54	0.257	0.305	− 0.048***
	(0.437)	(0.460)	0.000
Age 55 +	0.143	0.179	− 0.036***
	(0.350)	(0.384)	0.000
Age (Years)	42.162	44.694	− 2.532***
	(11.033)	(9.862)	0.000
Observations	167,256	187,761	355,017

Standard deviations in parentheses. The difference column shows the difference between MW female workers and female workers above the MW. The model used to predict the probability of being affected by the Minimum wage reform includes interactions among all the covariates presented in the table and provincial dummies

^*^p < 0.10, **p < 0.05, ***p < 0.01

**Table 8:** Main sample characteristics of females by survey wave

		2015		2019	
		(1)	(2)	(3)	(4)
		Treatment	Control	Treatment	Control
		mean	mean	Mean	mean
Female characteristics	*Educational Attainment Level*				
	Primary or less	0.033	0.012	0.141	0.044
	Secondary	0.831	0.453	0.697	0.324
	High school	0.101	0.101	0.118	0.146
	University	0.036	0.433	0.042	0.486
	*Municipality size*				
	Small	0.496	0.449	0.506	0.463
	Medium	0.381	0.368	0.392	0.370
	Large	0.123	0.183	0.102	0.167
	*Religion*				
	Catholic	0.644	0.681	0.667	0.685
	Other non-Catholic	0.356	0.319	0.333	0.314
	Age (Years)	36.3	48.7	37.8	48.9
	Immigrant	0.249	0.042	0.193	0.054
	Has children	0.726	0.812	0.748	0.812
*Partners characteristics*					
	Age (Years)	40.2	50.7	41.3	51.2
	*Partner education*				
	Primary or less	0.125	0.118	0.144	0.123
	Secondary	0.532	0.496	0.512	0.501
	High school	0.133	0.130	0.144	0.130
	University	0.209	0.256	0.201	0.246
	Immigrant Partner	0.149	0.035	0.117	0.041
	Observations	2,932	3,085	2,507	2,836

This table shows sample characteristics by survey wave and treatment status. Values represent sample means. The treated group consists of women with above median predicted probability of working in minimum wage jobs, while the control group includes women with below-median predicted probability. Sample: 11,360 women from Spanish Survey of Violence Against Women (2015: 6,017 women; 2019: 5,343 women)

**Table 9:** Covariates balance assessment: before and after inverse probability weighting

		Before IPW weighting		After IPW weighting	
		(1)	(2)	(3)	(4)
		Treatment	Control	Treatment	Control
		mean	mean	mean	mean
Female Characteristics	*Educational Attainment Level*				
	Primary or less	0.083	0.027	0.075	0.036
	Secondary	0.769	0.392	0.737	0.411
	High school	0.109	0.123	0.132	0.132
	University	0.039	0.459	0.056	0.421
	Municipality size				
	Small	0.223	0.203	0.226	0.215
	Medium	0.274	0.245	0.285	0.248
	Large	0.180	0.143	0.162	0.140
	Religion				
	Catholic	0.643	0.682	0.662	0.686
	Other non-Catholic	0.214	0.293	0.222	0.285
	No religion	0.143	0.024	0.116	0.029
	Age (Years)	36.9	48.7	40.3	48.3
	Immigrant	0.250	0.043	0.197	0.051
	Has children	0.726	0.813	0.753	0.813
Partners Characteristics					
	Age (Years)	40.15	50.99	43.4	50.5
	*Partner education*				
	Primary or less	0.125	0.118	0.144	0.123
	Secondary	0.532	0.496	0.512	0.501
	High school	0.133	0.130	0.144	0.130
	University	0.209	0.256	0.200	0.246
	Immigrant Partner	0.149	0.035	0.118	0.037
	Observations	5,439	5,921	5,439	5,921

This table shows covariate balance before and after inverse probability weighting (IPW). Values represent sample means by treatment status. Binary variables show proportions (0–1); age variables are in years. "Difference Change" shows change in absolute difference between groups (negative values indicate balance improvement). The treated group consists of women with above median predicted probability of working in minimum wage jobs, while the control group includes women with below-median predicted probability. Sample: 11,360 women from Spanish Survey of Violence Against Women (2015, 2019)

**Table 10:** Effect of minimum wage on intimate partner violence

	Model 1: DID		Model 2: DID-IPW	
Treated × Post	0.017**	0.019**	0.023*	0.027**
	(0.01)	(0.01)	(0.01)	(0.01)
Treated	0.005	− 0.010	0.000	− 0.012
	(0.01)	(0.01)	(0.01)	(0.01)
Post	− 0.019***	− 0.015	− 0.022***	− 0.020*
	(0.01)	(0.01)	(0.01)	(0.01)
Covariates	No	Yes	No	Yes
Control group mean IPV (%)	6.73	6.73	6.73	6.73
*N*	11,360	11,360	11,360	11,360

Robust standard errors clustered at provincial level in parentheses. Model 1 DID refer to Difference-in-Differences, and Model 2 DID-IPW refers to Double Robust Difference-in-Differences using Inverse Probability Weighting. Covariates used are: Age, Education, Immigrant, having a child, Municipality Size, Region of residence, age of the partner, education of the partner and immigrant partner. These covariates are defined in Table 2 Survey IPV 2015–2019

^*^p < 0.10, **p < 0.05, ***p < 0.01

## Data Availability

Submission ID: SERI-D-24-00130 “Economic policy and psychological violence: the hidden costs of Spain’s minimum wage reform” We use 2 datasets: Survey of Violence Against Women, years 2014–15 and 2019; and Muestra Continua de Vidas Laborales, 2018. Data from the Survey of Violence Against Women is publicly available and anyone can access them in https://violenciagenero.igualdad.gob.es/Macroencuesta2015/https://violenciagenero.igualdad.gob.es/macroencuesta2015/macroencuesta2019/. However, we are not allowed to share the Muestra Continua de Vidas Laborales. Authors can request access to latter data set. We will provide do files that will enable to replicate our results upon request.

## Appendix

See Tables 7, 8, 9, and 10.

## Footnotes

1 For instance, most of the research on Earned Income Tax Credit (EITC) focuses on women without a four-year college degree because the EITC expansion targeted low- to moderate-income working individuals and couples (Hoynes et al. 2013 and related literature).

2 Gender norms are known to persistently impact intimate partner violence in Europe as shown by González & Rodríguez-Planas (2020).

3 In Spain, annual earnings include 14 payments as there are two months (August and December) in which the payment doubles.

4 Figure 2 plots full-time equivalent wages rather than actual wages as explained in detail in the Data Section. This explains why there appears to be no workers below the minimum wage in this figure. Since we have converted all part-time wages to their full-time equivalent, workers who might be earning below the minimum wage in absolute terms (due to part-time status) are represented at their full-time equivalent rate, which would typically be at or above the minimum wage.

5 Prior to this change, starting in 2011, the surveys were conducted in person rather than by phone. This shift resulted in a smaller sample size but allowed for a more detailed and in-depth questionnaire. Sample sizes decreased from 20,552, 20,652 and 28,423 in 1999 to 2006, respectively, to 7,898 in 2010–11, 10,171 in 2014–15 and 9,568 in 2019. Despite being in person as in 2014–15 and 2019, the 2010–11 wave was substantially different in questionnaire design, response options, and measurement approach, which would make it methodologically unsound to combine with the 2014–2015 and 2019 waves.

6 There are 50 provinces in Spain.

7 We requested and were granted access the MCVL to the Dirección General de Ordenación de la Seguridad Social.

8 For part-time workers, we compute full-time equivalent monthly wages using a two-step procedure. First, we calculate hourly wages by dividing observed wages by the actual hours worked, which are derived from the worker's part-time coefficient (reported in the MCVL as a percentage of full-time hours). Second, we convert these hourly wages to full-time equivalent monthly earnings by multiplying by the standard workweek of 40 h and 52/12 weeks per month. We are aware that this approach does not allow us to know the exact monthly wage given that the MCVL does not have hours worked, but it is a good approximation to determine whether the worker is or not affected by the minimum wage reform.

9 This approach minimizes the sum of squared residuals while applying a penalty term that reduces some coefficients to zero, effectively selecting the most relevant predictors and preventing overfitting. To select the LASSO penalty parameter, we rely on the Bayesian Information Criterion (BIC), reflecting the specific demands of our application. In our setting, the model is trained on administrative wage data (MCVL) and applied to a separate survey dataset (SVAW). BIC imposes a stronger penalty on model complexity than cross-validation and is consistent in model selection under standard regularity conditions (Schwarz 1978; Ahrens et al. 2020). This preference for sparsity reduces the risk of overfitting to MCVL-specific features that may not generalize to the SVAW data. While cross-validation typically optimizes in-sample predictive accuracy, our objective is to obtain stable and transferable predictions across distinct data sources. In this context, BIC’s conservative selection is preferable, aligning with recent applications in economics that prioritize out-of-sample classification over within-sample fit (Mullainathan and Spiess 2017; Autor et al. 2021; Cengiz et al. 2022a, b).

10 We trained the model over the 80% of the total sample of women in the MCVL and used the 20% to select the measure predictive model capacity. The predictive model capacity was measure using the area under the ROC curve. For the two penalization methods (cross-validation and BIC) the area under the ROC curve was 0.72, and for the plugin method it was 0.71.

11 This median is defined using the pre-reform period sample of women in the SVAW sample.

12 As the survey was fielded between September 12 and December 1 2019, some of the violence may have occurred in the last quarter of 2018, prior to the minimum wage increase. This would under-estimate IPV in 2019.

13 Appendix Table 10 shows the key estimates of these regressions.

14 On average only 40% of the women in our sample are employed, compared to 70% of their partners.
